# Novel Aerogel Structure of β-Eucryptite: Featuring Low Density, High Specific Surface Area, and Negative Thermal Expansion Coefficient

**DOI:** 10.3390/gels11060440

**Published:** 2025-06-09

**Authors:** Haoren Ma, Sijia Liu, Jinyi Ren, Xiaochan Liu, Weiyi Zhang, Ying Zhu, Zhipeng Yuan, Jinxu Zhu, Xibin Yi

**Affiliations:** Shandong Key Laboratory of Advanced Glass Manufacturing and Technology, Advanced Materials Institute, Qilu University of Technology (Shandong Academy of Sciences), Jinan 250014, China; 17864099575@163.com (H.M.); a1244775883@163.com (J.R.); liuxiaochan@sdas.org (X.L.); zhangweiyi@sdas.org (W.Z.); zhuy@sdas.org (Y.Z.); yuanzhipeng@sdas.org (Z.Y.); 17864099610@163.com (J.Z.)

**Keywords:** β-eucryptite, aerogel, sol–gel method, negative thermal expansion, low cost

## Abstract

Traditional β-eucryptite (LiAlSiO_4_) is renowned for its unique characteristics of low thermal expansion and high temperature thermal stability, making it an ideal material for precision instruments and aerospace applications. In this study, β-eucryptite was fabricated into an aerogel structure through the sol–gel process and supercritical drying method and using alumina sol as a cost-effective precursor. The synthesized β-eucryptite aerogel demonstrated unique properties including a negative thermal expansion coefficient (−7.85 × 10^−6^ K^−1^), low density (0.60 g/cm^3^), and high specific surface area (18.1 m^2^/g). X-ray diffraction (XRD) and transmission electron microscopy (TEM) mutually corroborated the crystalline structure of β-eucryptite, with XRD confirming the phase purity and TEM imaging revealing well-defined crystal lattice characteristics. Combined nitrogen adsorption–desorption analysis and scanning electron microscopy observations supported the hierarchical porous microstructure, with SEM visualizing interconnected nanoporous networks and nitrogen sorption data verifying the porosity. The negative thermal expansion behavior was directly linked to the β-eucryptite crystal structure, as collectively validated by thermal expansion measurements. Additionally, Fourier transform infrared spectroscopy (FTIR) independently confirmed the aluminosilicate framework structure through characteristic vibrational modes. This research shows the innovation in the synthesis of β-eucryptite aerogel, especially its application potential in precision instruments and building materials that need low thermal expansion and high stability, and the use of aluminum sol as an aluminum source has simplified the preparation steps and reduced production costs.

## 1. Introduction

The discovery of negative thermal expansion (NTE) materials represents a significant breakthrough in materials science [1]. Traditional materials expand when heated, while NTE materials contract, making them crucial in modern technology [2,3]. NTE materials are particularly effective in high-precision environments where temperature fluctuations cause dimensional changes. They help maintain system stability and improve instrument accuracy and reliability. For instance, the key components of spacecraft and satellites will experience extreme temperature changes, and negative thermal expansion materials can reduce thermal stress to ensure the normal operation of the equipment [4]. Cao et al. [5] developed a method to reduce the thermal expansion coefficient by inserting NH_3_ into the voids of the framework structure to control the thermal expansion of ZrW_2_O_8_. The material exhibits a thermal expansion coefficient of −2.1 × 10^−6^ K^−1^, which effectively reduces thermal stress and enhances thermal stability, raising the phase transition temperature by approximately 50 K.

In microelectronics technology, NTE materials [6] such as β-eucryptite mica can reduce thermal expansion and contraction caused by heat generated in circuits and chips, thereby improving performance [7,8]. Miller et al. [9] study showed that adding NTE material can reduce thermal stress by 19.8%. NTE materials are also beneficial for construction as they reduce cracks caused by temperature fluctuations in concrete and improve the durability of buildings [10]. Ouyang et al. [11] proposed a novel cemented composite material doped with ZrW_2_O_8_, which can adjust the coefficient of thermal expansion (CTE) from 8.65 × 10^−6^ °C^−1^ to 2.48 × 10^−6^ °C^−1^. This composite material can be applied in cement-based materials to prevent structural or equipment failures and irreversible damage.

As shown in Table 1, common materials with negative thermal expansion coefficients generally suffer from poor stability, toxicity, and high cost. Among them, ZrW_2_O_8_ belongs to the cubic crystal system and has a three-dimensional network “open framework” structure. When heated, the [WO_4_] tetrahedrons in the framework rotate counterclockwise around the Zr–O–W bonds, causing lattice parameter contraction and achieving isotropic negative thermal expansion. However, it starts to decompose into ZrO_2_ and WO_3_ at temperatures exceeding 700 °C, leading to structural collapse. Sc_2_W_3_O_12_ has a cubic pyrochlore structure, featuring a highly symmetric distorted octahedral coordination environment in its lattice, which allows uniform contraction of lattice parameters as the temperature rises. Nevertheless, it is expensive and prone to oxidation. As the prototype of lithium aluminum silicate (LAS) [12], β-eucryptite is a rare natural substance with negative thermal expansion, as well as excellent thermal shock resistance, dielectric properties, and thermal stability [13]. By combining β-eucryptite mica with other materials, low-expansion or “zero”-expansion composite materials can be manufactured, thereby improving the volume stability [14,15]. T. Shimada and Khattab et al. [16,17] successfully prepared composite materials with coefficients of thermal expansion (CTEs) of 1.45 × 10^−6^ K^−1^ and −1.31 × 10^−6^ K^−1^ by combining yttria stabilized zirconia with 55% or 65% β-eucryptite mica. Based on the above, in the past few decades, it has attracted numerous researchers to conduct basic and technical research [18].

β-eucryptite has many excellent properties. However, its relatively high production cost, large density and high interfacial resistance limit its wide application. The expense issue makes it difficult to be promoted on a large scale. Moreover, the large density usually leads to a relatively small specific surface area, which has a negative impact on some material properties. Compared with other NTE materials [19,20], its expense-related drawback makes it less competitive among traditional NTE materials. The relatively small specific surface area reduces its effectiveness in battery applications due to fewer available active sites. Meanwhile, the large density will not only reduce its heat dissipation ability in precision instruments but also make the material face more difficulties in the process of processing and manufacturing. When grinding and shaping such high-density materials, more energy is required, and more durable tools are needed [21], which undoubtedly further increases the production expense and prolongs the production time. This study attempts to explore new precursors to control the expense and use new methods to increase the specific surface area and reduce the density, so as to expand the application scope of the materials.

Aerogel materials are famous for their excellent specific surface area, ultra-high porosity, large pore volume, and extremely low density [22,23]. These unique properties make aerogels have great application prospects in the fields of heat insulation, adsorption, energy storage, and catalysis [24]. Due to its transformative potential, aerogels are often called “future materials” and have attracted great attention in scientific research and industrial applications [25]. Generally, aerogels are synthesized by a sol–gel process combined with supercritical drying technology, which makes the formation of nanoscale pores possible. This structural design can effectively increase the specific surface area and reduce the density, making aerogel an ideal choice for various applications [26]. With the continuous progress of material science and engineering, the advantages brought by transforming traditional materials into aerogel structures have become increasingly remarkable. For example, traditional TiO_2_, when used as a catalyst, often fails to achieve the desired catalytic effect [27]. However, after being made into an aerogel structure, TiO_2_ exhibits higher catalytic performance. Liu et al. [28] prepared a composite aerogel by making TiO_2_ into an aerogel structure. The prepared composite aerogel has a high specific surface area of 287.3 m^2^/g and a pore volume of 0.72 cm^3^/g, which helps significantly improve the absorption ability of reactants and enables rapid intra-particle molecular transfer for visible light catalysis, and thus, the photocatalytic performance is greatly enhanced. Similarly, after SiO_2_ is made into aerogel structure, its density and weight are significantly reduced, its specific surface area is increased, and its thermal insulation performance is also enhanced [29]. Chen et al. [30] made SiO_2_ into an aerogel structure. The prepared SiO_2_ aerogel exhibited good thermal insulation performance, and its thermal conductivity at room temperature was only 0.025 W/(m·K). These improvements in structural design have led to a wider range of materials being classified as aerogels, thus opening up new innovative ways for the composition and application of aerogel materials.

In particular, integrating β-eucryptite into an aerogel structure provides a promising approach to enhance the practical application of β-eucryptite. As a lithium aluminosilicate renowned for its unique negative thermal expansion (NTE) properties, β-eucryptite mica holds enormous potential in fields such as aerospace and construction [31,32]. The core value of negative thermal expansion materials lies in their ability to counteract the thermal expansion of other materials through thermal contraction, thereby reducing thermal stress. The high-specific-surface-area aerogel structure enhances thermal management capabilities in the following ways: 1. Regulation of thermal conduction pathways: The porous network of aerogels increases the scattering effect of thermal conduction, reducing the overall thermal conductivity. This leads to more uniform distribution of thermal stress under temperature gradients, avoiding deformation caused by local overheating. 2. Efficiency of heat exchange with the environment: A larger specific surface area means a greater contact area between the material and the surrounding medium, potentially accelerating heat dissipation or absorption to rapidly balance temperatures and reduce the accumulation of thermal stress. This makes it more suitable for applications in precision instruments and aerospace. However, its applications are constrained by issues such as cost, porosity, and density, often rendering it non-competitive. Transforming β-eucryptite into an aerogel effectively mitigates these limitations. The mesoporous network of β-eucryptite aerogels not only enhances their thermal stability and elasticity but also improves their adaptability to high-stress environments and thermal cycles, thus expanding their application in the construction field. Furthermore, the aerogel structure significantly increases the specific surface area of β-eucryptite, which enhances its internal active sites, making it an ideal choice for the microelectronics sector.

The cost issue is another challenge that troubles the application of β-eucryptite garnet. The precursors of traditional aerogels can be divided into organic and inorganic. The common organic aluminum precursors include tertbutyl aluminum and isopropanol aluminum. The use of organic aluminum sources involves complex reaction processes, and the hydrolysis and condensation rates are difficult to control [33]. Traditional inorganic aluminum sources, such as aluminum chloride and aluminum nitrate, although more affordable, introduce various ions that affect the experimental results [34]. Different from the traditional preparation method of aerogels, this new method bypasses the hydrolysis process and directly conducts polycondensation [35]. This innovative method simplifies the synthesis process, reduces production costs, and minimizes ion interference to the greatest extent possible. At the same time, in order to conduct a control experiment to verify whether the prepared performance has an impact, this study also used AlCl_3_·6H_2_O as an aluminum source for the preparation of β-eucryptite garnet. Inspired by the method of directly preparing aerogel from silica sol, aluminum sol was prepared with appropriate concentration and then directly became a gel and was combined with other functional components [36].

Transforming β-eucryptite into an aerogel structure has obvious performance advantages. First, the inherent negative thermal expansion characteristics of β-eucryptite pyroxene are effectively preserved. Second, the large specific surface area of the aerogel structure enhances the interaction with the surrounding environment. The high porosity and interconnected pore structure further enhance its thermal shock resistance, enabling it to withstand rapid temperature changes without structural degradation. These unique properties make β-eucryptite aerogel a multifunctional material, which will have better application prospects in aerospace, microelectronics, and construction fields.

## 2. Results and Discussion

**Figure 1:**
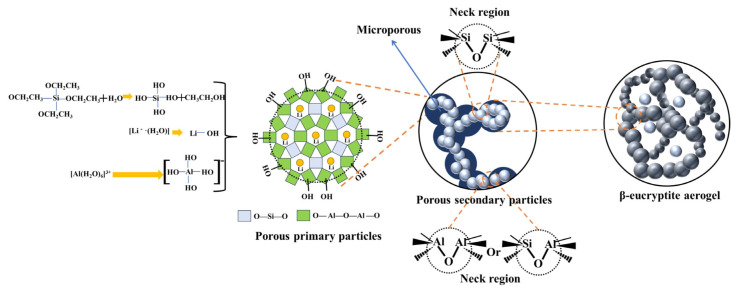
Schematic diagram illustrating the formation mechanism of LASA.

Figure 1 illustrates the network formation process in the aerogel structure. Initially, TEOS reacted with water to form [Si(OH)_4_], while LiCl dissociated into Li^+^ and Cl^−^ ions in aqueous solution. The Li⁺ ions interacted strongly with water molecules, forming hydrated complexes such as [Li(H_2_O)_X_]^+^, which further reacted to produce lithium hydroxide (LiOH) [37]. Simultaneously, Al^3+^ ions from the aluminum sol formed [Al(H_2_O)_6_]^3+^ complexes that lost protons, leading to the formation of hydroxylated aluminum species like [Al(OH)_4_]^−^. These species underwent polymerization, eventually forming primary particles that then aggregated to form secondary particles. The secondary particles grew larger and interconnected to form the final aerogel network, with each secondary particle representing an amplified version of the primary particles. During the process of network formation, three different types of connection modes were formed between the particles: 1. Si–O–Si: This region formed between silicon-based primary particles ([Si(OH)_4_]) and involved the condensation of Si–O–Si bonds. These linkages contributed to the overall silicon network within the aerogel. 2. Al–O–Al: This region formed between aluminum-based primary particles ([Al(OH)_4_]^−^) and involved the condensation of Al–O–Al bonds. These linkages were critical for integrating the aluminum component of the aerogel structure. 3. Si–O–Al: This region formed between silicon and aluminum particles and involved Si–O–Al linkages. This type of connection was essential for forming a mixed silicon-aluminum network, which was characteristic of lithium aluminosilicate aerogels. The formation of secondary particles played a key role in the aerogel’s structure. As the primary particles condensed and polymerized, many secondary particles began to link together, eventually forming the three-dimensional network characteristic of LASA aerogels. This process of secondary particle formation and aggregation continued until a stable network structure was established. After heat treatment, the secondary particles underwent further consolidation, and the aerogel transformed into the final β-eucryptite phase, enhancing its mechanical strength and internal porosity. The crystallization into β-eucryptite stabilized the network through Li–O bonds, creating a stable and highly porous structure with high surface area. Thus, the network formation, including the development of primary and secondary particles and the three distinct neck regions (Si–O–Si, Al–O–Al, and Si–O–Al linkages), was essential for the aerogel’s final structure and properties.

**Figure 2:**
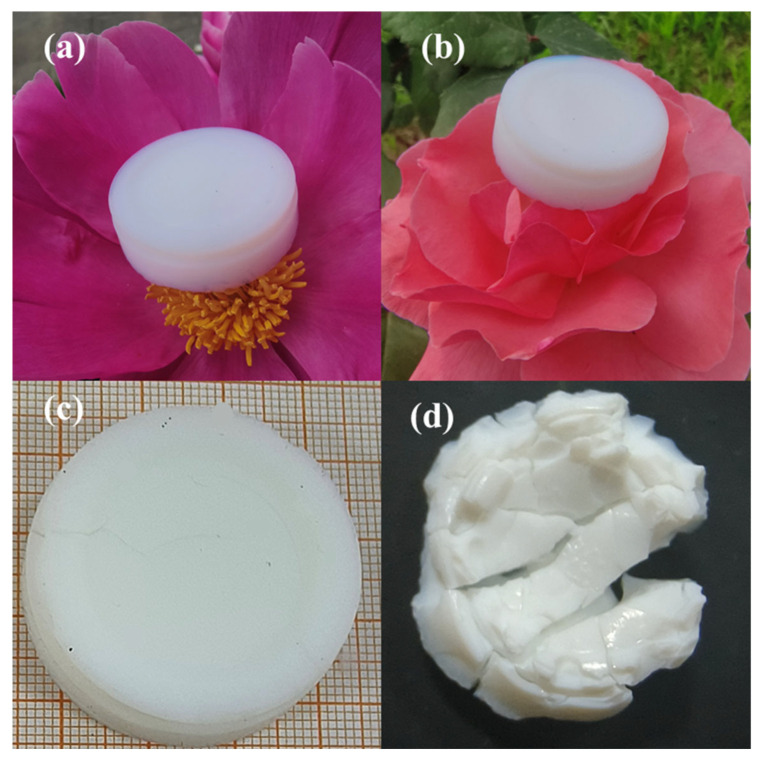
(**a**,**b**) The optical images of LASA-1-25 placed on flower stamens. (**c**) The optical image of LASA-1-750. (**d**) The optical image of LASA-1-1000.

Figure 2a,b show the optical images of the as-prepared LASA-1-25 samples. It can be observed that their mass was extremely low, such that they could be positioned on flower petals without inducing any deformation. The bulk sintered densities were determined by the Archimedes’ method. Figure 2c is the sample after calcination at 750 °C, which remained nearly intact with low linear shrinkage—a 12% reduction compared with its state at 25 °C and a density of 0.36 g/cm^3^. Figure 2d is the β-eucryptite aerogel after calcination at 1000 °C, and significant linear shrinkage was observed, which was 52% lower compared with that at 25 °C. Its density increased to 0.60 g/cm^3^, but it was only one-fourth of the density of conventional β-eucryptite (2.398 g/cm^3^) [14].

**Figure 3:**
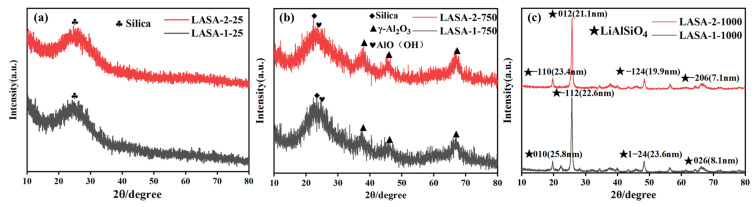
(**a**) X-ray diffraction pattern of LASA after calcination at as dried. (**b**) X-ray diffraction pattern of LASA at 750 °C. (**c**) X-ray diffraction pattern of LASA at 1000 °C.

Figure 3 shows the X-ray diffraction patterns of a dried β-eucryptite aerogel and samples after heat treatment at 750 °C and 1000 °C. It can be seen that the broad diffraction peaks of LASA-1 and LASA-2 at 25° after drying corresponded to the mixed peak of amorphous SiO_2_. After calcination at 750 °C for 1 h, similarly, the wide diffraction peak of LASA-1 and LASA-2 at 25° corresponded to the mixing peak of amorphous SiO_2_, while the wide peaks at 37°, 46°, and 68° corresponded to γ-alumina because the transformation of aluminum aerogel from boehmite phase to γ-Al_2_O_3_ occurred at about 700–800 °C, and the broad peak at 27° corresponded to boehmite (AlO(OH), PDF No. 83-2384). Upon elevating the thermal treatment temperature to above 1000 °C, it could be distinctly observed through Figure 3b that both LASA-1 and LASA-2 samples exhibited characteristic diffraction peaks of the β-hematite crystalline phase at 20°, 26°, and 49°. This suggested that the crystallization of the β-eucryptite crystal structure did not occur at temperatures below 750 °C, whereas calcination at 1000 °C facilitated the acquisition of the β-eucryptite component with a higher degree of crystallinity. The crystal forms of LASA-1 and LASA-2 were almost identical at various temperatures, and lithium garnet crystal forms appeared after calcination at 1000 °C.

**Figure 4:**
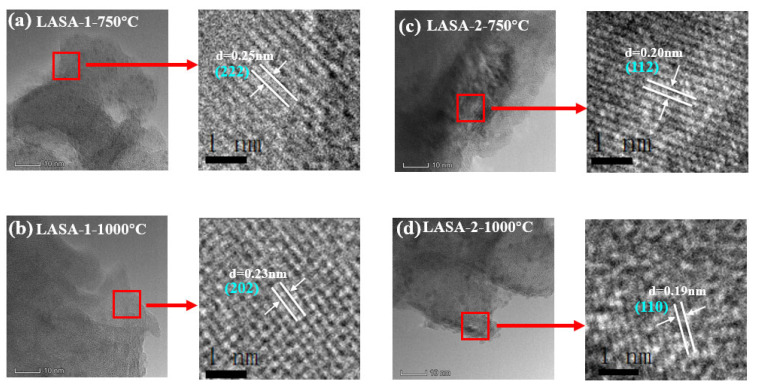
HR-TEM analysis: (**a**) LASA-1-750, (**b**) LASA-2-750, (**c**) LASA-1-1000, and (**d**) LASA-2-1000.

Figure 4 illustrates the high-resolution transmission electron microscopy (HR-TEM) analysis of LASA-1 and LASA-2 samples calcined at 750 °C and 1000 °C, highlighting the evolution of their crystalline structures. The observed lattice fringes and corresponding d-spacings revealed the effects of calcination temperature on the formation of crystal phases, which were further supported by XRD results and standard reference data. In Figure 4a, the HR-TEM image of LASA-1 calcined at 750 °C showed lattice fringes with a d-spacing of 0.25 nm, corresponding to the (222) plane. Combined with XRD analysis, this indicated that the sample was primarily in the γ-Al_2_O_3_ phase at this temperature, reflecting the partial crystallization of the material. Similarly, Figure 4c displays the HR-TEM image of LASA-2 calcined at 750 °C, where the d-spacing of 0.20 nm corresponds to the (112) plane of the γ-Al_2_O_3_ phase [38]. Upon increasing the calcination temperature to 1000 °C, the crystalline structure evolved significantly, transitioning to the β-eucryptite phase, as confirmed by XRD and the comparison with standard reference cards. In Figure 4b, the HR-TEM image of LASA-1 calcined at 1000 °C shows lattice fringes with a d-spacing of 0.23 nm, corresponding to the (202) plane of β-eucryptite. This indicates that the higher calcination temperature facilitated grain growth and the development of a well-defined β-eucryptite crystal structure. Similarly, Figure 4d shows the HR-TEM image of LASA-2 calcined at 1000 °C, with a d-spacing of 0.19 nm, attributed to the (110) plane of β-eucryptite. These observations confirmed that both samples underwent a phase transition from γ-Al_2_O_3_ to β-eucryptite at 1000 °C. The HR-TEM analysis, combined with XRD results, demonstrated that the calcination temperature played a crucial role in determining the crystalline phase of the samples. At 750 °C, both LASA-1 and LASA-2 exhibited the γ-Al_2_O_3_ phase, while at 1000 °C, they transitioned to the β-eucryptite phase, as evidenced by the identified crystal planes and corresponding d-spacings.

**Figure 5:**
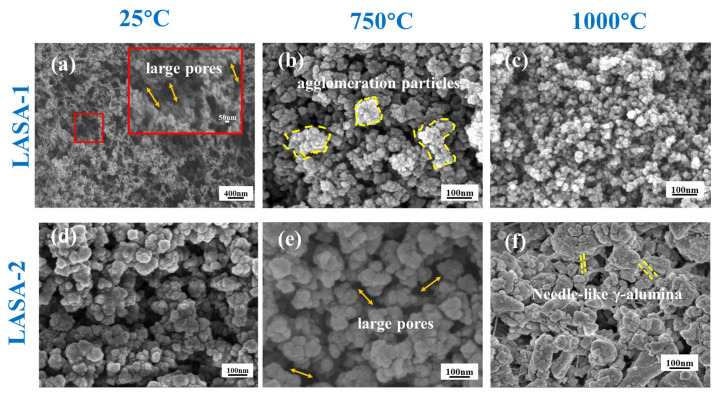
SEM analysis: (**a**) LASA-1-25, (**b**) LASA-1-750, (**c**) LASA-1-1000, (**d**) LASA-2-25, (**e**) LASA-2-750, and (**f**) LASA-2-1000.

Figure 5 shows the typical scanning electron microscopy (SEM) images of LASA-1 and LASA-2 samples under different heat treatment temperatures. The as-dried LASA composite aerogels exhibited a highly porous colloidal structure, characteristic of the typical pearl chain-like morphology of aerogels. Figure 5a,d reveal the presence of macropores larger than 100 nm. The significant difference between LASA-1 and LASA-2 at 25 °C could be attributed to the choice of precursor. LASA-1 was prepared using AlCl_3_ 6H_2_O as the precursor, which resulted in a more uniform, compact network due to the simpler gelation process and tighter binding of the aluminum chloride particles. On the other hand, LASA-2, which was synthesized using aluminum sol, showed a more disordered morphology at 25 °C, likely due to the colloidal nature of aluminum sol. This caused uneven dispersion and particle agglomeration, leading to a less uniform structure. Despite these differences in initial morphology, both samples exhibited similar internal porosity. In Figure 5b,e, a significant change in morphology was evident after heat treatment at 750 °C for 2 h. Both samples showed particle agglomeration due to the collapse of the internal structural framework at high temperatures, resulting in reduced internal porosity. Furthermore, Figure 5c,f show the morphology of LASA after calcination at 1000 °C for 2 h. After calcination, relatively obvious blocky solids could be seen inside. This was because the crystal form of β-eucryptite appeared at 1000 °C. In Figure 5f, the presence of needle-like γ-alumina whiskers can be observed. This formation was primarily due to the differences in the ratio of aluminum to silicon compared with LASA-1. The addition of aluminum sol in LASA-2 introduced more aluminum, leading to the formation of γ-alumina whiskers, which was not as pronounced in LASA-1 prepared with AlCl_3_·6H_2_O. The needle-like structures were a direct result of the enhanced aluminum content and the different gelation behavior of the aluminum sol, which encouraged the growth of γ-alumina whiskers under heat treatment.

**Figure 6:**
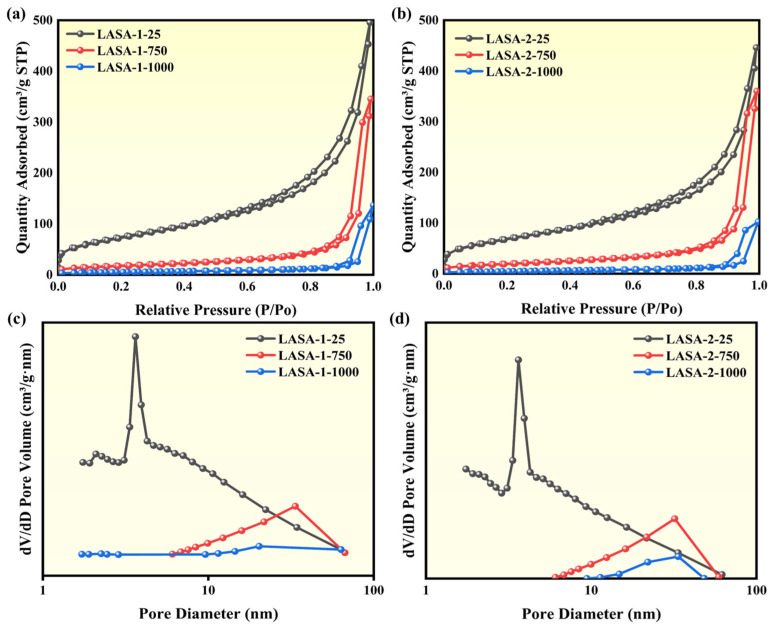
(**a**) N_2_ adsorption–desorption isotherms of LASA-1 at different temperatures. (**b**) N_2_ adsorption–desorption isotherms of LASA-2 at different temperatures. (**c**) Pore size distribution plots of LASA-1 at different temperatures. (**d**) Pore size distribution plots of LASA-2 at different temperatures.

Figure 6 presents the N_2_ adsorption–desorption isotherms and pore size distribution curves of dried and heat-treated LASA samples. Figure 6a,b show the N_2_ adsorption–desorption isotherms of LASA samples heat-treated at different temperatures. According to IUPAC classification, these isotherms were of type IV, characterized by H3 hysteresis loops, indicating the presence of irregular pore structures [39], consistent with the typical porosity characteristics of aerogel materials. These isotherms did not exhibit a clear saturation adsorption plateau, further indicating that the internal pore structure of the samples was complex and diverse. Figure 6a,b show that as the heat treatment temperature increased, the adsorption capacity of the LASA samples decreased significantly. This phenomenon could be attributed to the partial shrinkage and collapse of internal pores caused by the increase in temperature, leading to a decrease in adsorption capacity. Additionally, when the relative pressure P/P0 approached 1, the adsorption capacity of the LASA samples increased sharply, indicating the presence of larger pore structures in these samples, which may significantly impact overall adsorption performance. Figure 6c,d show the pore size distribution of LASA-1 and LASA-2 samples under different heat treatment temperatures.

According to Table 2, we can see that the pore size of LASAs increased with temperature. For example, LASA-1 had an average pore size of 8.75 nm at 25 °C but reached 24.41 nm at 1000 °C with increasing temperature. For LASA materials exhibiting negative thermal expansion, this may be due to the fact that the frame may initially shrink with heating, but at sufficiently high temperatures, other factors such as sintering of the matrix or instability of the frame (especially when using aluminum sol) may cause pore expansion rather than shrinkage. Further observation revealed that after high-temperature treatment at 1000 °C, the pore volume of LASA-2 decreased compared with LASA-1, although the reduction was not significant. This phenomenon may be attributed to the reduced strength of the framework formed by the aluminum sol used in LASA-2 compared with AlCl_3_·6H_2_O, which was consistent with the results observed in SEM. The use of aluminum sol may have led to the relative instability of the framework structure after high-temperature treatment, slightly affecting its adsorption performance. Additionally, the specific surface area of lithia-silica aerogels decreased with increasing temperature due to gradual structural collapse within the aerogel matrix. Even after treatment at 1000 °C, the specific surface area remained above 18.1 m^2^/g. Notably, after 750 °C treatment, the specific surface area still retained 70.9 m^2^/g, which was 10 times higher than that of traditional β-eucryptite materials (7 m^2^/g) [16].

**Figure 7:**
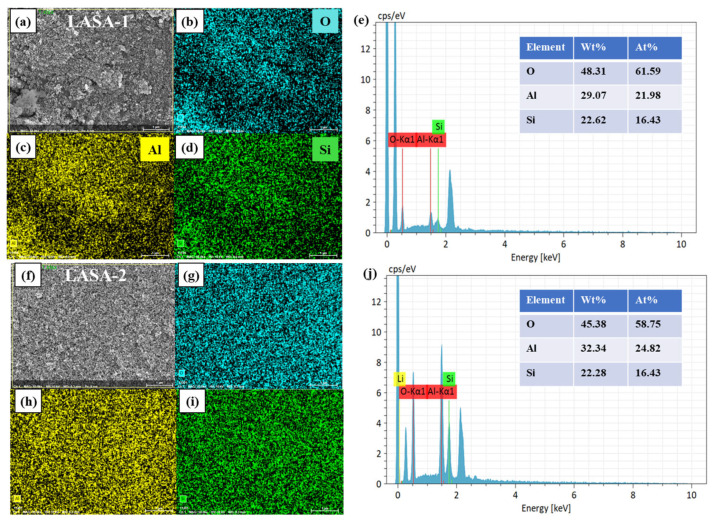
(**a**) SEM image of LASA-1 at 10,000× magnification. (**b**–**d**) Elemental distributions of LASA-1. (**e**) Elemental distribution mapping image of LASA-1. (**f**) SEM image of LASA-2 at 10,000× magnification. (**g**–**i**) Elemental distributions of LASA-2. (**j**) Elemental distribution mapping image of LASA-2.

As shown in Figure 7, the element distribution in LASA aerogel was observed by energy dispersive X-ray spectroscopy (EDX). Due to the low energy of the Li element, it could not be detected, so only the distribution of O, Al, and Si elements was available. Figure 7a,f show the scanning electron microscopy (SEM) image of the LASA sample after 1000 °C heat treatment, magnified 10,000 times, as well as the elemental distribution and EDX analysis results of the observed area. In the selected area, the distribution of three elements in LASA samples prepared by two different precursors appeared to be uniform, with elements concentrated along the skeleton and sparse in the pore area. According to the EDX results, the atomic contents of O, Al, and Si in LASA-1 were 61.59%, 21.98%, and 16.43%, respectively, while the atomic contents of O, Al, and Si in LASA-2 were 58.75%, 24.82%, and 16.43%, respectively. The main peak corresponded to O, Al, and Si, which were close to the experimental theoretical values.

**Figure 8:**
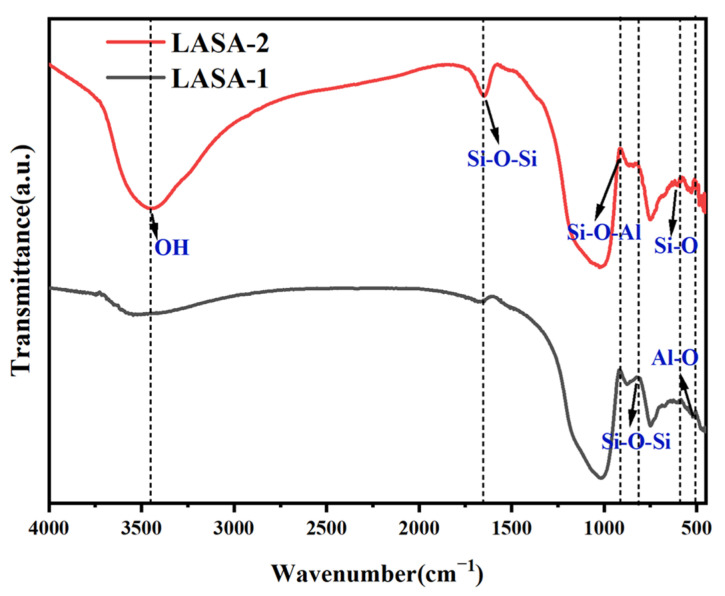
FTIR of LASA-1 and LASA-2 after heat treatment at 1000 °C.

Figure 8 displays the FT-IR spectra of LASA, revealing several characteristic absorption bands that provide insights into the chemical structure of the aerogels. The broad absorption band at 3500 cm^−1^ corresponded to OH stretching vibrations, which were associated with the hydrolysis and condensation processes during sol–gel synthesis. The prominent peak at 1800 cm^−1^ was attributed to the asymmetric stretching vibrations of Si–O–Si bonds, indicating the presence of a typical silica network and confirming the integrity of the SiO_2_ framework in the aerogel. The Si atom band corresponding to the stretched bonding motion was observed at around 1048 cm^−1^ against its tetrahedral oxygen cage. Another significant peak around 930 cm^−1^ was due to the stretching vibrations of Si–O–Al bonds, which suggested the incorporation of aluminum into the silica framework and the formation of Al–O–Si linkages. This peak confirmed the successful doping of aluminum, leading to the formation of aluminosilicate structures within the aerogel. The peak near 790 cm^−1^ was associated with symmetric stretching vibrations of Si–O–Si bonds, further confirming the presence of a robust silica network. The region between 500 and 700 cm^−1^ contained several absorption bands that were related to bending vibrations of metal–oxygen bonds, such as Al–O and Si–O, which corroborated the mixed oxide composition and the potential formation of β-eucryptite. Although the vibrations of lithium–oxygen (Li–O) bonds were generally difficult to detect directly in FT-IR spectra due to the low vibrational energy of Li–O bonds, the presence of lithium could be indirectly inferred. This was manifested by slight shifts in the peak positions and changes in the intensities of the bands associated with Si–O–Si and Si–O–Al bonds. The incorporation of lithium into the silica network typically caused slight shifts in the positions of the Si–O–Si and Si–O–Al stretching vibration peaks, particularly in the regions around 800–1200 cm^−1^. These shifts were often subtle but could be attributed to changes in the local bonding environment due to the presence of lithium ions. The intensity of the peaks corresponding to Si–O–Si and Si–O–Al bonds may also change slightly due to the influence of lithium on the bonding interactions within the silica network. The indirect evidence of lithium incorporation was clearly observed through these spectral shifts and intensity changes.

**Figure 9:**
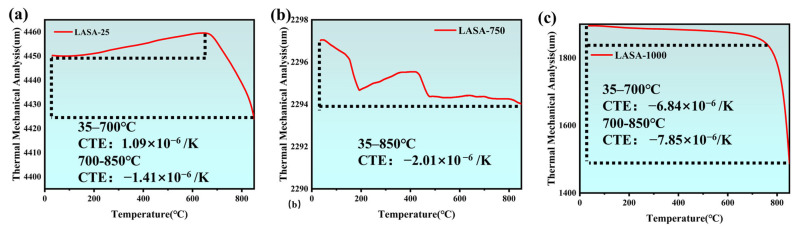
Thermal expansion analysis: (**a**) thermal expansion plot of LASA-25, (**b**) thermal expansion plot of LASA-750, and (**c**) thermal expansion plot of LASA-1000.

Figure 9 presents the CTE results of β-eucryptite aerogel samples (LASA-2-25, LASA-2-750, and LASA-2-1000) formed after heat treatment at different temperatures. By analyzing the thermal expansion coefficients of the samples over different temperature ranges (CTE), the negative expansion behavior characteristic of β-eucryptite could be revealed. The thermal expansion behavior of the LASA-2-25 sample within the range of 35–700 °C exhibited an initial positive expansion coefficient (1.09 × 10^−6^ K^−1^). This initial positive expansion behavior could be attributed to the incomplete formation of the β-eucryptite phase in the material, leading to typical thermal expansion as the temperature increased. However, as the temperature further increased, the material gradually began to transform into the β-eucryptite phase, leading to lattice contraction and consequently exhibiting a trend toward a negative expansion coefficient at higher temperatures. This process suggested that at 700 °C, the β-eucryptite crystalline phase had not yet fully formed and remained in a transitional state. For the LASA-2-750 sample calcined at 750 °C, the coefficient of thermal expansion (CTE) curve initially showed a downward trend, gradually rose at higher temperatures, and finally decreased. Within the range of 35–850 °C, the material exhibited a negative expansion coefficient (−2.01 × 10^−6^ K^−1^). This phenomenon could be explained by the fact that the sample calcined at 750 °C had partially transformed into the β-eucryptite phase but had not yet completed this transformation. Therefore, the sample still exhibited some positive expansion behavior at lower temperatures, while at higher temperatures, as the β-eucryptite phase gradually formed, the material began to exhibit negative expansion characteristics. Figure 9c shows the thermal expansion behavior of the LASA-2-1000 sample after calcination at 1000 °C. Within the temperature range of 35–850 °C, the sample exhibited a negative expansion coefficient of (−7.85 × 10^−6^ K^−1^), indicating a consistent negative expansion. This suggested that under the calcination conditions of 1000 °C, the β-eucryptite phase had fully formed, displaying its characteristic negative expansion properties. This phenomenon is typically associated with the anisotropic lattice structure of β-eucryptite, particularly its lattice contraction behavior at high temperatures. This stable negative expansion behavior of the material further confirms the complete transition to β-eucryptite and aligns with its unique crystalline structure characteristics.

## 3. Conclusions

In this study, β-eucryptite aerogels with high porosity, stable structure, and distinctive negative thermal expansion were successfully synthesized using a combined sol–gel and supercritical ethanol drying method. The main findings were as follows:

1. The β-eucryptite aerogel exhibited low density (0.60 g/cm^3^) and a high specific surface area of 18.1 m^2^/g and maintained a mesoporous structure with an average pore width of 29.64 nm at room temperature. This confirmed the formation of a stable, interconnected aerogel framework.

2. HR-TEM and XRD analyses confirmed γ-Al_2_O_3_ phase at 750 °C (d = 0.25/0.20 nm) and β-eucryptite formation at 1000 °C (d = 0.23/0.19 nm) in LASA samples, demonstrating temperature-dependent phase evolution and grain growth.

3. After heat treatment, the aerogels demonstrated phase transitions essential for thermal stability. Specifically, at 1000 °C, the β-eucryptite crystalline phase was fully established, maintaining a stable nano-structured skeleton and achieving a negative thermal expansion coefficient of −7.85 × 10^−6^ K^−1^.

4. Utilizing aluminum sol as a precursor simplified the synthesis process and significantly reduced costs, enhancing the feasibility for large-scale applications of β-eucryptite aerogels in high-precision and high-temperature environments.

In conclusion, this work addressed key limitations of traditional β-eucryptite materials by introducing an efficient synthesis route for aerogels with superior thermal properties. Future research will focus on enhancing mechanical strength to expand the aerogels’ application in structural components without compromising their insulation and thermal stability.

## 4. Materials and Methods

### 4.1. Materials

The study used aluminum chloride hexahydrate (AlCl_3_·6H_2_O, 97%, Aladdin Reagent Shanghai Co., Ltd., Shanghai, China), lithium chloride monohydrate (LiCl·H_2_O, 99.5%, McLean Biochemical Technology Shanghai Co., Ltd., Shanghai, China), industrial aluminum sol (23%, self-prepared, alcohol-based), and tetraethyl orthosilicate (TEOS, Aladdin Reagent, Sinopharm Chemical Reagent Shanghai Co., Ltd., Shanghai, China) as precursors; polyacrylic acid (PAA, 97%, Aladdin Reagent Shanghai Co., Ltd.) as a dispersant; 1,2-epoxypropane (PO, 99.0%, Aladdin Reagent Shanghai Co., Ltd.) as a proton scavenger; deionized water (99.99%, self-prepared) as a hydrolysis agent; and absolute ethanol (99.9%, Aladdin Reagent Shanghai Co., Ltd.) as a solvent. The alumina sol was an inexpensive colloidal system, formed by fine particles of positively charged aluminum oxyhydroxide monohydrate (AlOOH) dispersed in a solution. It was primarily prepared from 23% aluminum oxide and 77% ethanol, with a pH value of approximately 6. The impurity content was ≤0.02%, which was caused by the residual trace amounts of monovalent acid peptizer remaining during the preparation process. The specific preparation process is explained as follows.

### 4.2. Method

**Figure 10:**
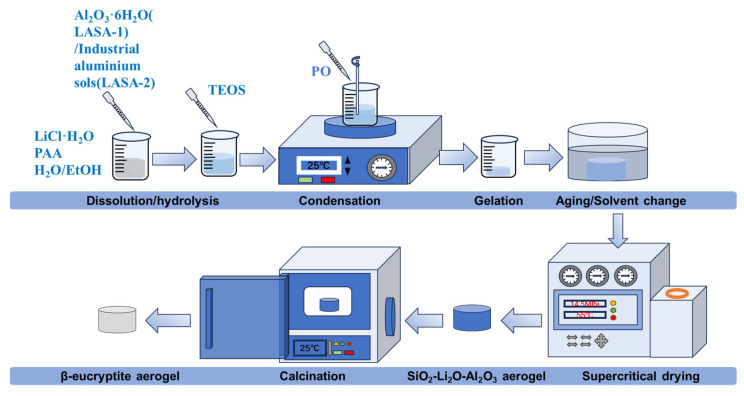
Schematic diagram of the preparation process.

As shown in Figure 10, using LiCl·H_2_O as the raw material, Li-Al-Si oxide composite gels were synthesized via an inorganic dispersion synchronous sol–gel method. The precursors included lithium chloride monohydrate (LiCl·H_2_O), aluminum chloride hexahydrate (AlCl_3_·6H_2_O), industrial aluminum sol, and tetraethyl orthosilicate (TEOS). Polyacrylic acid (PAA) served as the dispersant, deionized water served as the hydrolyzing agent, absolute ethanol served as the solvent, and 1,2-propylene oxide (PO) served as the proton scavenger for gelation.

The synthesis procedure was as follows: First, LiCl·H_2_O and PAA were added, followed by the synchronous addition of H₂O and EtOH with stirring until complete dissolution. Subsequently, AlCl_3_·6H_2_O or industrial aluminum sol was introduced, and the mixture was stirred for 5 min. Then, TEOS was added, and the stirring was continued for 1 h. Finally, PO was added, and the mixture was stirred for an additional 5 min. The molar ratio of the components was maintained as LiCl·H_2_O:AlCl_3_·6H_2_O:industrial aluminum sol:TEOS:H_2_O:EtOH:PAA:PO = 1:0.75:50:14.72:1:1.44:0.04:9. The composite gel was poured into a mold and aged at 25 °C for one week, during which anhydrous ethanol was added to the mold for solvent exchange. The exchanged gel was then dried using supercritical CO_2_ drying for 4 h to prepare Li_2_O-Al_2_O_3_-SiO_2_ composite aerogels with different ratios. The aerogels were placed in a muffle furnace, heated at a rate of 5 °C/min to 1000 °C, calcined for 2 h, and naturally cooled to 25 °C to obtain β-eucryptite aerogels (LASA). The β-eucryptite aerogel samples prepared using AlCl_3_·6H_2_O as a precursor were designated as LASA-1, while those prepared using industrial aluminum sol were designated as LASA-2.

**Figure 11:**
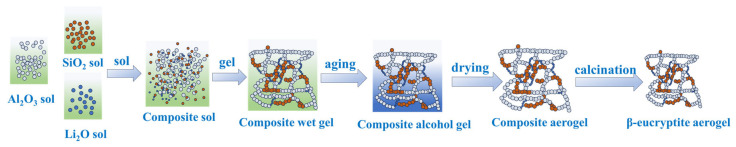
Structural evolution of β-eucryptite aerogels.

Figure 11 illustrates the polymerization mechanism for the formation of LASA composite aerogels. The process began with the hydrolysis and condensation of LiCl·H_2_O, AlCl_3_·6H_2_O (industrial aluminum sol), and TEOS in the presence of PAA as a dispersant and gelation agent. This resulted in the formation of a Li-Al-Si oxide composite gel. After aging and solvent exchange, the gel underwent supercritical CO_2_ drying to preserve its structure. The dried gel was then calcined at 1000 °C, causing structural reorganization and the formation of the β-eucryptite phase. The differences in precursor materials (AlCl_3_·6H_2_O for LASA-1 and aluminum sol for LASA-2) led to variations in the gelation behavior and the resulting morphology, with LASA-1 showing a more uniform structure and LASA-2 exhibiting a more disordered network.

### 4.3. Characterization

The specific surface area and pore size distribution of the aerogel were tested by the porosity analyzer (Micromeritics APSP 2460, Micromeritics Instrument Corporation, Norcross, GA, USA) and calculated by using the Barrett–Joyner–Halenda (BJH) analytical method. The crystal structure of the aerogel was characterized by an X-ray diffractometer (XRD, Rigaku Ultima, Rigaku Corporation, Tokyo, Japan) and Cu-Kα radiation (λ = 1.54 Å). The sample was ground into powder, loaded onto a glass substrate, and pressed onto a flat surface, and the scanning rate was 0.1 s/step. Fourier transform infrared spectroscopy (FT-IR, Thermo Scientific Nicolet iS20, Thermo Fisher Scientific Inc., Waltham, MA, USA) was used to measure the chemical bonding of aerogels in transmission mode. The test wave number was 400–4000 cm^−1^. The microstructure of the aerogels was measured by scanning electron microscopy (SEM, Zeiss Gemini 300, Carl Zeiss AG, Oberkochen, Germany) under 10 kV conditions. The internal structure of the aerogels was observed and measured using transmission electron microscopy (TEM, JEM 2100, JEOL Ltd., Tokyo, Japan) under a voltage of 200 kV. The thermal expansion coefficient of the aerogels was calculated using a dilatometer. The heating rate was 5 °C/min up to 850 °C (NETZSCH DIL402 Expedis Supreme, NETZSCH-Gerätebau GmbH, Selb, Germany).

## Figures and Tables

**Table 1:** Common negative expansion coefficient materials.

Material	Chemical Formula	Negative Expansion Coefficient Range (10^−6^ K^−1^)	Specific Surface Area (m^2^/g)	Drawbacks
Eucryptite silicate	LiAlSiO_4_	−5 to −10	6	High cost, high density, low specific surface area
Zirconium tungstate	ZrW_2_O_8_	−8.7 to −8.9	5	Poor thermal stability, decomposes at high temperatures
Zirconium vanadate	ZrV_2_O_7_	−6 to −9	3	Poor chemical stability, easily hydrolyzed
Scandium tungstate	Sc_2_W_3_O_12_	−7 to −10	2	High cost, complex preparation process
Tantalum niobate	TaNbO_5_	−5 to −8	3	High cost, complex preparation
Cuprous oxide	Cu_2_O	−2 to −5	4	Easily oxidized, poor chemical stability
Cadmium cyanide	Cd(CN)_2_	−17 to −18	2	Toxic, high environmental and health risks

**Table 2:** Microstructure properties of LASAs with different heat-treated temperatures.

Sample	LASA-1	LASA-2
25 °C		
Specific surface area (m^2^/g)	262.4	243.9
Pore volume (cm^3^/g)	0.76	0.68
Average pore width (nm)	8.75	8.41
750 °C		
Specific surface area (m^2^/g)	64.8	70.9
Pore volume (cm^3^/g)	0.53	0.55
Average pore width (nm)	21.78	23.54
1000 °C		
Specific surface area (m^2^/g)	19.4	18.1
Pore volume (cm^3^/g)	0.17	0.15
Average pore width (nm)	24.41	29.64

## Data Availability

The data presented in this study are available on request from the corresponding authors.
